# The Book World of Medicine and Science

**Published:** 1899-03-04

**Authors:** 


### The Book World of Medicine and Science.

The Year Book of Treatment for 1899. (London: Cassell and Co. 1899. Price 7s. 6d.)

This ever-welcome volume fully maintains its position as a complete summary of the progress of the year in regard to all that concerns treatment. It is, however, much more than a mere *résumé* of what has been printed during the preceding twelve months, and the help it gives to the practitioner is something very different from what he could obtain by looking through the bound volume of his journals, for the contents of the "Year Book" fully support the claim made upon its title page that it is a "critical review." And herein lies its value. Good men, writing each on his own subject, give, in about 450 pages, a well-balanced sketch of the advances in treatment which have been made and a well-thought-out appreciation of the various therapeutic proposals which have been thrown out. The vast extent of medical journalism and the growing number of observers in all parts of the world who are now in the habit of recording their experiences, make it absolutely necessary that some such annual s'ocktaking of progress should be undertaken, and this cou'd not be better done than it is in the series of annual volumes to which the one before us forms the most recent addition. Following the chapter on "Diseases of the Lungs," we find in this issue a special one on "The Open-Air Treatment of Phthisis," by Dr. Burton-Fanning, in which all that is known on the subject is well summarised and descriptions are given of many of the places in which the new treatment is carried out. Much interest is added to this chapter by the various illustrations which are given showing the arrangements for the patients at the different resorts alluded to. Altogether it is a most useful and labour-saving book for anyone who wishes to keep at hand, for ready reference, a permanent record of all that is most important in each year's work.

Sanatoria for Consumptives in Various Parts of the World, together with an Exposition of the Open Air or Hygienic Treatment of Phthisis. By F. Rufenacht Walters, M.D., M.R.C.P., with an introduction by Sir Richard Douglas Powell, Bart., M.D., F.R.C.P. (London: Swan Sonnenschein and Co. 1899. Price 10s. 6d. net.)

This is a book the publication of which is most opportune, for not only has the author gathered together a vast amount of information which, but for his laborious inquiries, would have been quite inaccessible to ordinary readers, but this information is exactly of the sort which is required by those on whom the responsibility is cast of deciding what is to be done with persons suffering from consumption. The work commences with a short preface by Sir Douglas Powell, in which he generally endorses the conclusions of the author. Then come chapters dealing with the climates most suitable for consumptives, describing the objects of sanatoria, and giving a general outline of sanatorium treatment. After these we have a series of most useful chapters, essential to all who have to do with the construction and management of such institutions, presenting details as to cost, plan, furniture, disinfection, staff, and many other things in which sanatoria differ on the one hand from hospitals and on the other from hotels, and finally a careful and pretty full description is given of the leading sanatoria which exist in various parts of the world, this part of the book being freely illustrated with plans and other drawings. The main importance of the book lies in the large number of facts that have been collected together by the author; for the reader soon finds that the practice in regard to many of the details varies greatly in different places, both as regards the construction of the sanatoria and the treatment of the patients. It is too early as yet to dogmatise or lay down fixed rules; the field is open for improvement on present practice in many directions, and what is wanted by those who are engaged in the work is full information as to what is being actually done in different parts of the world, and this is just what the book gives. At the same time, although the major part of the book is descriptive, we must not omit to say a word in praise of those parts in which Dr. Rufenacht Walters discusses the principles of treatment, for he has evidently thought deeply on the subject, and the views he puts forward are expressed in such a judicial and impartial manner as to carry conviction to the minds of those who read. The last chapter, in which a comparison is drawn between the provision made in Germany and in England for the reception of consumptives, is rather disheartening reading, and shows how far ahead of ourselves some countries are in this matter. "Six years ago, when Germany's first sanatorium for the poor was erected, England was unrivalled in her provision for the consumptive poor. There were at that time no such institutions in any other part of the Continent, whereas in the British Isles there were some 17 special hospitals and nursing homes in existence, with over 1,100 beds, besides other institutions open to consumption, though not exclusively devoted to them. Since that time, however, great strides have been made in Germany, so that she will very soon be far ahead of England, both in the number and the character of her institutions of this kind. . . . Germany has at the present time about 20 sanatoria in working order, with an aggregate of nearly 1,300 beds, while in the near future there will be nearly double the number of sanatoria, with over 2,400 beds. . . . Moreover, the majority of these sanatoria are exclusively for consumption and in extremely well chosen spots . . . whereas the British institutions are mostly urban, and shared with sufferers from bronchitis and heart disease." As for sanatoria for paying patients, the comparison is ludicrous. In Germany they provide nearly 1,200 beds, against 55 in England, the latter figure including such sanatoria as are being built as well as those [illegible] already exist.

Physician's Book for Private Formulæ: with Posological Table. (Bristol: John Wright and Co. Price 2s. and 2s. 6d.)

This is a little memorandum book arranged on an alphabetical plan, in which to enter such formulæ as may be found useful in practice. In addition to the blank pages there is a posological table according to the British Pharmacopœia, a list of external applications from the same source, a list of poisons and their treatment, and other useful information. The book is quite of waistcoat-pocket size, and is beautifully bound in compressed leather.

### BOOKS RECEIVED.

The Scientific Press.
"The London Water Supply: A Retrospect and a Survey." By Richard Sisley, M.D., Lond., M.D. (State Med.)

John Wright and Co.
"Physician's Book for Private Formulæ; with Posological Table."

